# The association of family history of herpes zoster and the risk of incident herpes zoster: the SHEZ Study

**DOI:** 10.1265/ehpm.21-00020

**Published:** 2022-05-28

**Authors:** Keiko Kinumaki, Hironori Imano, Yukiko Takao, Yoshinobu Okuno, Yasuko Mori, Hideo Asada, Koichi Yamanishi, Hiroyasu Iso

**Affiliations:** 1 Public Health, Department of Social Medicine, Osaka University Graduate School of Medicine, Osaka, Japan; 2 Research Institute for Microbial Diseases, Osaka University, Japan; 3 Division of Clinical Virology, Kobe University Graduate School of Medicine, Hyogo, Japan; 4 Department of Dermatology, Nara Medical University School of Medicine, Nara, Japan; 5 The Research Foundation for Microbial Diseases of Osaka University, Osaka, Japan

**Keywords:** Herpes zoster, Family history, History of HZ, Incidence, Prospective cohort study

## Abstract

**Background:**

We investigated whether family histories of herpes zoster (HZ) are associated with the risk of incident HZ in a Japanese population.

**Methods:**

A total of 12,522 Japanese residents aged ≥50 years in Shozu County participated in the baseline survey between December 2008 and November 2009 (the participation rate = 72.3%). They were interviewed at baseline by research physicians regarding the registrants’ history of HZ. A self-administered questionnaire survey was conducted to evaluate the potential confounding factors. 10,530 participants without a history of HZ were followed up to ascertain the incidence of HZ during 3-years follow-up until the end of November 2012 with Japanese nationals. We estimated hazard ratios (HRs) of incident HZ according to first-degree family histories using the Cox proportional hazard regression after adjusting for age, sex, and other potential confounding factors.

**Results:**

Compared to no HZ history of each family member, a history of brother or sister was associated with a higher risk of incident HZ while histories of father and mother were not. The multivariable HR (95%CI) of incident HZ for a history of brother or sister was 1.67 (1.04–2.69). When comparing to no family histories of all first-degree relatives, the multivariable HRs (95%CIs) were 1.34 (0.77–2.34) for a history of brother or sister alone, but 4.81 (1.78–13.00) for a history of mother plus brother or sister. As for the number of family histories, the multivariable HRs (95%CIs) were 1.08 (0.76–1.54) for one relative (father, mother, or brother or sister) and 2.75 (1.13–6.70) for two or more relatives.

**Conclusion:**

Family histories of mother plus brother or sister and two or more first-degree relatives were associated with a higher risk of incident HZ.

## Graphical Abstract

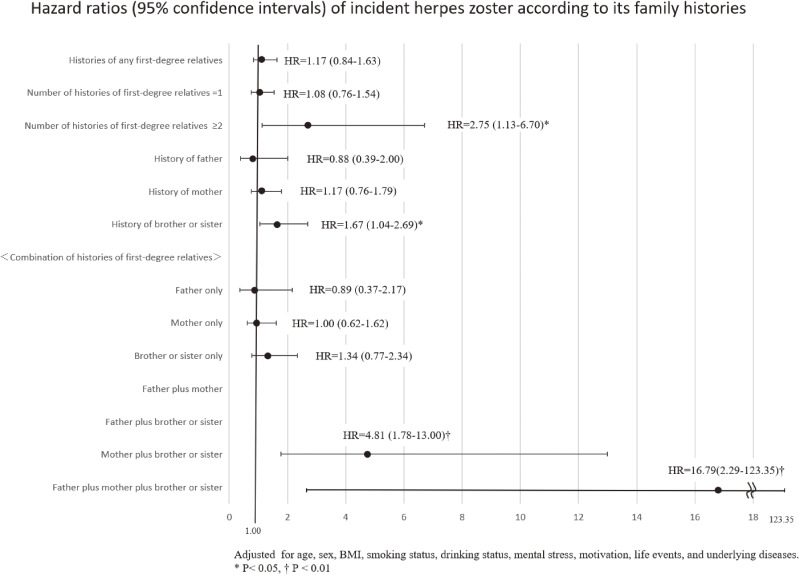

## Background

Herpes zoster (HZ) is a painful disease caused by varicella-zoster virus (VZV) infection. Patients with HZ usually experience painful vesicular rashes with erythema, which generally take 3–4 weeks to heal [1]. According to a 2017 investigation by the Ministry of Health, Labour and Welfare, the number of patients with HZ was approximately 13,900 per year in Japan [2]. VZV causes varicella in childhood as an initial stage of infection; the VZV then latently infects the sensory nerve ganglia of the host, where the virus is reactivated, usually by reduction of immune function [3].

A family history of HZ has been reported as a risk factor for HZ [4–10] for which the Interleukin (IL)-10 promotor polymorphism among Koreans [11] and apolipoprotein E-ε4 polymorphism among Caucasian women [12] were associated with susceptibility of HZ. However, previous studies came from retrospective case-control studies in the United States, Europe, Iran, and China. No prospective study has investigated the association between a family history of HZ and the risk of incident HZ. Retrospective case-control studies are susceptible to selection, recall, interviewer biases and failure of control for potential confounding variables which could distort the association.

Potential confounding variables other than age and sex were lower body mass index (BMI) [13], smoking [14], drinking [15], mental stress [16], motivation [16], negative life event [16], underlying diseases such as hypertension, hyperlipidemia, diabetes mellitus [17], cancer [18], and connective tissue diseases [19].

The goal of the present study was to examine whether first-degree family histories of HZ and the number of these family histories were positively associated with the risk of incident HZ in a free-living general population.

## Methods

### Study subjects

A prospective cohort study was undertaken in Shozu County, Kagawa Prefecture, consisting mainly of Shodoshima and Toyoshima Islands with a census population of 33,782, of which 32.8% were 65 years or older on July 1, 2008. The detailed methods of this study have been described elsewhere [20].

The eligible study subjects were 19,058 (8,424 men and 10,634 women) Japanese residents aged ≥50 years on October 1, 2008. The provisional registration was performed for 12,896 persons by confidential mail, and those who completed the informed consent were formally registered. Eventually, 12,522 (5,587 men and 6,935 women) were registered between December 2008 and November 2009 (the overall participation rate among total residents = 72.3%).

**Fig. 1:**
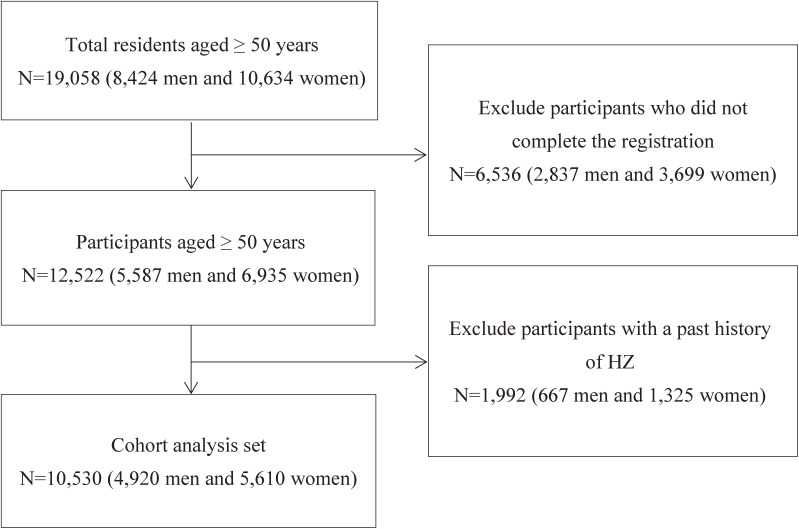
Flow chart of the selection for study participants

We excluded 1,992 (667 men and 1,325 women) participants who had a past history of HZ, the remaining 10,530 (4,920 men and 5,610 women) were included in the analysis. The participants’ enrollment process is shown in Figure 1.

### Data collection and follow-up

At baseline survey, an interview was conducted by research physicians to inquire about the participants’ history of HZ and when it had occurred, and whether they had visited a clinic or hospital. A self-administered questionnaire was used to evaluate factors including age, height, weight, and current health status (smoking status, drinking status, mental stress, motivation, life events, and underlying diseases such as hypertension, hyperlipidemia, diabetes mellitus, connective tissue disease, leukemia, and cancer), and family histories of first-degree relatives (father, mother, brother or sister) of HZ.

Telephone surveys were conducted every four weeks to confirm the presence or absence of rash, pain, history of contact with patients with varicella, and admission to a clinic or hospital. Participants with symptoms suspicious for HZ, including rash had been asked to visit a clinic or hospital, and underwent blood tests. We directed most of all participants to consult a physician soon after developing symptoms in the monthly telephone assessments.

The duration of the follow-up was between April 2009 and November 2012.

### Statistical analysis

Differences in baseline characteristics according to the presence or absence of family histories of HZ were examined using the analysis of variance for mean values and chi-square test for frequency. The crude, age- and sex-adjusted, and multivariable HRs and 95% CIs of incident HZ were calculated according to the family histories using a Cox proportional hazard regression model, adjusting for age, sex, and other confounding variables.

The family histories of HZ were categorized into the presence versus absence of each family member, i.e., father, mother, and brother or sister, and the presence of father only, mother only, brother or sister only, father and mother, father and brother or sister, mother and brother or sister, and all of them versus the absence of all relatives. The number of family histories of HZ was grouped into none, one, and two or more of histories for father, mother and brother or sister.

The confounding factors for the multiple adjustment were BMI (kg/m^2^) calculated as the body weight divided by the square of the height, smoking status (never, former, and current smoker of 1–9, 10–19 and ≥20 cigarettes per day), drinking status (never, former, and current drinkers of <23, 23–45 and ≥46 g of ethanol per day), perceived mental stress (very high to high, moderate, and low), motivation (very high to high, moderate, and low), negative life events (no or yes), and underlying diseases (hypertension, hyperlipidemia, diabetes mellitus, cancer, and connective tissue diseases). All statistical analyses were performed using SAS for Windows (version 9.4; SAS Inc., Cary, NC, USA). P-values for statistical tests were two-tailed, and values <0.05 were considered statistically significant.

## Results

Table 1 shows the baseline characteristics according to family histories of HZ. Father’s history was positively associated with younger ages, mental stress, life events, and fewer underlying diseases. Mother’s history was positively associated with female sex, younger ages, BMI, mental stress, motivation, and fewer underlying diseases. A history of brother or sister was positively associated with female sex, non-smoking, non-drinking and more underlying diseases. The number of family history of HZ was positively associated with younger ages, non-drinking, and fewer underlying diseases.

**Table 1:** Baseline characteristics according to family histories of herpes zoster.

	**Total participants**	**Father’s history ** **of HZ**		**P for differences**	**Mother’s history ** **of HZ**		**P for differences**	**Brother’s or sister’s history of HZ**		**P for differences**	**Any relatives**			**P for differences**

		**No**	**Yes**		**No**	**Yes**		**No**	**Yes**		**0**	**1**	**≥2**	
Number	10530	10310	220		9885	645		10204	326		9407	1058	65	
Women, %	53.3	53.2	56.8	0.287	53.0	57.4	0.032	52.8	68.1	<0.001	52.4	60.4	58.5	<0.001
Age, year	67.8 (10.6)	68.0 (10.6)	59.3 (6.8)	<0.001	68.2 (10.6)	61.2 (7.6)	<0.001	67.8 (10.7)	66.8 (9.1)	0.082	68.4 (10.7)	62.6 (8.5)	60.4 (7.4)	<0.001
Body mass index, kg/m^2^	22.8 (3.3)	22.8 (3.3)	22.7 (3.0)	0.601	22.8 (3.3)	23.1 (3.2)	0.028	22.8 (3.3)	23.1 (3.5)	0.104	22.8 (3.4)	23.0 (3.2)	22.9 (3.5)	0.061
Smoking status														
Non-smoker, %	57.7	57.6	63.2	0.021	57.5	61.2	0.004	57.4	66.9	<0.001	57.1	62.7	66.2	<0.001
Ex-smoker, %	22.3	22.4	15.5		22.5	19.4		22.3	21.8		22.6	20.0	13.9	
Current smoker, %	18.7	18.7	21.4		18.7	19.4		19.0	11.4		18.9	17.3	20.0	
Drinking status														
Non-drinker, %	50.3	50.3	51.8	0.293	50.3	51.2	<0.001	50.1	57.4	0.016	50.0	52.7	55.4	<0.001
Ex-drinker, %	6.1	6.1	5.0		6.2	3.3		6.1	4.9		6.3	4.0	4.6	
Current drinker, %	42.2	42.2	43.2		42.0	45.6		42.4	37.7		42.1	43.4	40.0	
Mental stress														
Very high to high, %	18.4	18.2	29.1	<0.001	17.9	26.4	<0.001	18.4	19.6	0.086	17.6	25.0	26.2	<0.001
Moderate, %	53.1	53.1	54.6		53.1	54.0		53.0	56.1		52.9	55.3	49.2	
Low, %	27.1	27.3	16.4		27.6	19.7		27.2	24.2		27.9	19.8	24.6	
Motivation														
Very high to high, %	69.4	69.4	69.1	0.159	69.2	71.2	0.006	69.4	66.9	0.058	69.3	70.8	60.0	0.001
Moderate, %	27.2	27.2	27.7		27.2	27.6		27.1	31.3		27.1	27.6	36.9	
Low, %	2.0	1.9	3.2		2.0	1.2		2.0	1.8		2.0	1.6	3.1	
Life events, %	10.3	10.1	17.7	<0.001	10.2	11.3	0.358	10.3	8.9	0.412	10.1	11.7	12.3	0.212
Underlying diseases, %	42.8	43.1	27.3	<0.001	43.2	36.4	0.001	42.6	50.3	0.005	43.3	39.1	33.9	0.012

Age and BMI showed mean values (standard deviations).

Analysis of variance was used for means and chi-square test was used for proportions.

Table 2 indicates crude, age- and sex-adjusted, and multivariable HRs and 95% CIs of incident HZ according to family histories for each first-degree relative and its combination, and the number of family histories of HZ. Compared to no history of each family member, a history of brother or sister was associated with the risk of incident HZ while histories of father and mother were not. The respective multivariable HRs (95%CIs) of incident HZ were 1.67 (1.04–2.69), 0.88 (0.39–2.00), and 1.17 (0.76–1.79). When comparing to no family histories of first-degree relatives, the multivariable HRs (95%CIs) were 1.34 (0.77–2.34) for a history of brother or sister alone, but 4.81 (1.78–13.00) for a history of mother plus brother or sister. The multivariable HRs were not calculated for histories of father plus mother, father plus brother or sister, and father plus mother plus brother or sister because of no or one case of HZ. As for the number of family histories, the multivariable HRs (95%CI) was 1.08 (0.76–1.54) for one relative (father, mother, or brother or sister) and 2.75 (1.13–6.70) for two or more relatives.

**Table 2:** Hazard ratios of incident herpes zoster according to family histories of HZ

**Family history of HZ**	**No of participants**	**No of cases**	**Total ** **person-** **years**	**Incidence (per 1000 person-years)**	**Crude ** **HR (95%CI)**	**Age- and sex-adjusted ** **HR (95%CI)^a^**	**Multivarible ** **HR (95%CI)^b^**
Histories of any first-degree relatives							
No	9407	300	27627.1	10.9	1.00	1.00	1.00
Yes	1123	41	3366.2	12.2	1.12 (0.81–1.56)	1.21 (0.87–1.69)	1.17 (0.84–1.63)
Number of histories of first-degree relatives							
0	9407	300	27627.1	10.9	1.00	1.00	1.00
1	1058	36	3175.3	11.3	1.04 (0.73–1.46)	1.11 (0.78–1.57)	1.08 (0.76–1.54)
≥2	65	5	190.9	26.2	2.40 (0.99–5.81)	2.71 (1.12–6.58)*	2.75 (1.13–6.70)*
History of father							
No	10310	335	30327.2	11.0	1.00	1.00	1.00
Yes	220	6	666.1	9.0	0.82 (0.36–1.83)	0.93 (0.41–2.10)	0.88 (0.39–2.00)
History of mother							
No	9885	318	29056.8	10.9	1.00	1.00	1.00
Yes	645	23	1936.5	11.9	1.09 (0.71–1.66)	1.21 (0.79–1.86)	1.17 (0.76–1.79)
History of brother or sister							
No	10204	323	30032.5	10.8	1.00	1.00	1.00
Yes	326	18	960.8	18.7	1.74 (1.08–2.80)*	1.67 (1.04–2.68)*	1.67 (1.04–2.69)*
Combination of histories of first-degree relatives							
None	9407	300	27627.1	10.9	1.00	1.00	1.00
Father only	185	5	560.9	8.9	0.82 (0.34–2.00)	0.95 (0.39–2.31)	0.89 (0.37–2.17)
Mother only	589	18	1773.4	10.1	0.94 (0.58–1.50)	1.04 (0.64–1.69)	1.00 (0.62–1.62)
Brother or sister only	284	13	841.0	15.5	1.42 (0.82–2.48)	1.35 (0.77–2.35)	1.34 (0.77–2.34)
Father plus mother	23	0	71.1	-	-	-	-
Father plus brother or sister	9	0	27.8	-	-	-	-
Mother plus brother or sister	30	4	85.7	46.7	4.30 (1.60–11.54)†	4.78 (1.78–12.84)†	4.81 (1.78–13.00)†
Father plus mother plus brother or sister	3	1	6.3	158.3	14.61 (2.05–104.08)†	17.70 (2.48–126.51)†	16.79 (2.29–123.35)†

^a^Adjusted for age and sex.

^b^Adjusted further for BMI, smoking status, drinking status, mental stress, motivation, life events, and underlying diseases.

*P < 0.05, †P < 0.01

## Discussion

In our community-based prospective cohort study of Japanese men and women aged ≥50 years living in Shozu County, a history of brother or sister was associated with a higher risk of incident HZ compared to no history of the siblings. When comparing to no family histories of the first-degree relatives, a history of mother plus brother or sister, and two or more number of family histories were associated with a higher risk of incident HZ.

A study for 504 cases and 523 controls of Americans matched by age, sex, and race reported that the ORs (95%CIs) of HZ were 4.50 (3.15–6.41) for a history of single blood relative and 13.77 (5.85–32.39) for that of multiple blood relatives, compared to no family history [4]. The following report of that study of 1103 cases and 523 controls showed the corresponding ORs (95%CIs) of 5.24 (3.79–7.23) and 17.15 (7.50–39.18), respectively [5]. A study for 389 cases and 511 controls of Americans matched by age, sex, immune compromise and vaccination statuses reported that the ORs (95%CIs) of HZ was 1.87 (1.34–2.60) for a history of the first-degree relatives compared to no family history [6]. A study for 217 cases and 200 controls of Iranians matched by age and sex showed that the OR (95%CI) of HZ were 4.91 (2.73–8.85) for a history of the first-degree relatives and 4.77 (2.16–10.54) for a history of the second-degree relatives compared to no each history [7]. A study for 656 cases and 656 controls of Americans matched by age, sex, and zoster vaccination reported OR (95%CI) of 1.17 (0.82–1.65) for a history of parents and 1.80 (1.16–2.79) for a history of siblings compared to no history of each relative [8]. A study for 250 cases and 500 controls of French matched by age and sex reported OR (95%CI) of 1.70 (0.96–3.01) for a history of father, 1.89 (1.24–2.88) for a history of mother, 2.87 (1.70–4.81) for a history of brother or sister compared to none of each history [9]. The stronger association for a history of brother or sister in the above two studies was consistent with our result. A Chinese population-based case-control study of 277 cases and 678 controls matched by age reported ORs (95%CI) of 2.4 (1.4–4.3) for a history of first-degree relatives compared to no family history [10]. To our best knowledge, this is the first to show significant associations of family histories and the number of family histories with the risk of incident HZ.

The strength of the present study included a fairly-high participation rate, systematic ascertainment of HZ cases, and the confirmation of HZ diagnosis by polymerase chain reaction detection of VZV [20]. However, our study had several limitations. First, the participants’ family histories of HZ were obtained from self-reports and were not validated by family records. A possible explanation for the lack of association between a history of father or mother and the risk of HZ may be its insufficient recall compared to a history of brother or sister due to the generational difference. Second, the number of incident HZ cases was small in the family history of HZ especially among the combination of histories of first-degree relatives to detect by sufficient statistical power. Finally, we cannot ignore the impact of residual confounding factors such as a history of varicella.

## Conclusion

Family histories of mother plus brother or sister and two or more first-degree relatives were associated with a higher risk of incident HZ among the free-living Japanese population.

## Abbreviations

CI: confidence interval
HR: hazard ratio
HZ: herpes zoster
SHEZ study: Shozu herpes zoster study
VZV: varicella-zoster virus
BMI: body mass index
OR: odds ratio
HR: hazard ratio

## Declarations

### Data availability statement

The data will not available to the others except for a academic scientists with investigator support after the approval of the study committee.
